# The structure function of the death domain of human IRAK-M

**DOI:** 10.1186/s12964-014-0077-3

**Published:** 2014-12-07

**Authors:** Jiangfeng Du, Gerry AF Nicolaes, Danielle Kruijswijk, Miranda Versloot, Tom van der Poll, Cornelis van ’t Veer

**Affiliations:** Department of Biochemistry, Cardiovascular Research Institute Maastricht, Maastricht University, Maastricht, The Netherlands; Center for Experimental and Molecular Medicine, Academic Medical Center, University of Amsterdam, Meibergdreef 9, Amsterdam, 1105 AZ The Netherlands

**Keywords:** IRAK-M, Inflammation, Death domain, Structure-function, TLR

## Abstract

**Background:**

IRAK-M is an inhibitor of Toll-like receptor signaling that acts by re-directing IRAK-4 activity to TAK1 independent NF-κB activation and by inhibition of IRAK-1/IRAK-2 activity. IRAK-M is expressed in monocytes/macrophages and lung epithelial cells. Lack of IRAK-M in mice greatly improves the resistance to nosocomial pneumonia and lung tumors, which entices IRAK-M as a potential therapeutic target. IRAK-M consists of an N-terminal death domain (DD), a dysfunctional kinase domain and unstructured C-terminal domain. Little is known however on IRAK-M’s structure-function relationships.

**Results:**

Since death domains provide the important interactions of IRAK-1, IRAK-2 and IRAK-4 molecules, we generated a 3D structure model of the human IRAK-M-DD (residues C5-G119) to guide mutagenesis studies and predict protein-protein interaction points. First we identified the DD residues involved in the endogenous capacity of IRAK-M to activate NF-κB that is displayed upon overexpression in 293T cells. W74 and R97, at distinct interfaces of the IRAK-M-DD, were crucial for this endogenous NF-κB activating capacity, as well as the C-terminal domain (S445-E596) of IRAK-M. Resulting anti-inflammatory A20 and pro-inflammatory IL-8 transcription in 293T cells was W74 dependent, while IL-8 protein expression was dependent on R97 and the TRAF6 binding motif at P478. The IRAK-M-DD W74 and R97 binding interfaces are predicted to interact with opposite sides of IRAK-4-DD’s. Secondly we identified DD residues important for the inhibitory action of IRAK-M by stable overexpression of mutants in THP-1 macrophages and H292 lung epithelial cells. IRAK-M inhibited TLR2/4-mediated cytokine production in macrophages in a manner that is largely dependent on W74. R97 was not involved in inhibition of TNF production but was engaged in IL-6 down-regulation by IRAK-M. Protein-interactive residues D19-A23, located in between W74 and R97, were also observed to be crucial for inhibition of TLR2/4 mediated cytokine induction in macrophages. Remarkably, IRAK-M inhibited TLR5 mediated IL-8 production by lung epithelial cells independent of W74 and R97, but dependent on D19-A23 and R70, two surface-exposed regions that harbor predicted IRAK-2-DD interaction points of IRAK-M.

**Conclusion:**

IRAK-M employs alternate residues of its DD to inhibit the different inflammatory mediators induced by varying TLRs and cells.

**Electronic supplementary material:**

The online version of this article (doi:10.1186/s12964-014-0077-3) contains supplementary material, which is available to authorized users.

## Introduction

Interleukin-1 receptor-associated kinase M (IRAK-M) is a member of the IRAK protein family [1], a family of proteins that is crucially involved in signaling initiated by the cytokines IL-1 and IL-18 and in Toll-like receptor activation [2]. Activation of the receptors leads to dimerization of the adaptor protein MyD88, and subsequent recruitment of IRAK-4 and other IRAK’s to form multimers (myddosomes) by homo- and heteromeric interactions of the death domains present in these IRAK’s [3,4]. Binding and phosphorylation events triggered by IRAK-4 result in hyper- and auto-phosphorylation of IRAK-1 and formation of IRAK-1/TRAF-6 complexes which dissociate from the receptor to activate TAB2/3 and TAK-1 [5]. TAB/TAK/TRAF6 activity leads to IκBα phosphorylation and ubiquitination, culminating in nuclear factor-κB (NF-κB) activation and transcription of inflammatory genes [5]. IRAK-2 hyperphosphorylation and TAB/TAK/TRAF6 activity leads to specific IRAK-2 dependent mRNA stabilization and translational control of pro-inflammatory mediators [6-8]. All IRAK family members mediate activation of NF-κB and MAPK [1] and the phenotype of IRAK-1, IRAK-2 and IRAK-4 deficient mice or cells is one of decreased production of inflammatory mediators [5]. In contrast, IRAK-M deficient mice or cells display an increased inflammatory response [9], illustrating the essentially different function of IRAK-M.

Structurally, IRAK-M consists of a kinase domain (KD) flanked by an N-terminal death domain (DD) involved in binding to other IRAK family members and an unstructured C-terminal domain (CTD) with a TRAF6 binding motif [1,10]. IRAK-1 and IRAK-4 contain active kinase domains, but IRAK-M and IRAK-2 lack the critical active site aspartate residue and appear devoid of kinase activity [1,10]. It has been reported that IRAK-M inhibits IRAK signaling by binding to the IRAK-1/IRAK-4 dimer assembled on the receptor-bound MyD88, thereby preventing IRAK-1/TRAF6 downstream signaling [9]. However, also other mechanisms have been put forward by which IRAK-M may inhibit inflammation in a more active manner, such as through IRAK-M dependent stabilization of MKP-1 [11] and down-regulation of the non-canonical NF-κB pathway [12]. Only recently it was shown that murine IRAK-M is redundant with IRAK-1/2 with respect to NF-κB activation through a unique IRAK-4/IRAK-M mediated MEKK3 pathway [13]. This pathway specifically induces IRAK-M dependent transcription of negative regulators such as A20, IκBα, SOCS-1 and SHIP [13]. Furthermore, IRAK-M was shown to inhibit the IRAK-2 dependent mRNA stabilization/translation of cytokines and chemokines [13]. Increased host responses caused by lack of IRAK-M are favorable for the clinical outcome in bacterial pneumonia [14-16], but also in tumor models [17] and bone marrow transplantation [18] which implies that inhibition of IRAK-M might have therapeutic potential. Different from the other IRAK’s, IRAK-M expression is rather restricted to certain cell types such as monocytes/macrophages and lung epithelial cells [1,19] and IRAK-M is up-regulated under inflammatory conditions and associated with LPS tolerance [9,20].

Zhou *et al.* [13] showed that the conserved W74 residue in the DD of murine IRAK-M was crucial for the interaction with IRAK-4 and NF-κB activation. Here we show that both the DD as well as the CTD of IRAK-M are important for the endogenous NF-κB activation capacity of human IRAK-M upon overexpression in 293T cells. We investigated the structure-function relationships of the human IRAK-M DD by mutagenesis of predicted protein interaction sites. We identified at least 3 sites on the DD of IRAK-M which are involved in IRAK-M’s function. Two of these, located on opposite sides of the death domain and constellated by W74/Q78/F18 and R97/Y105 respectively, are predicted to interact with different sides of the IRAK-4 DD. Both sites and the interactive area in between these sites formed by the surfacing stretch D19 to A23 were observed to be involved in the capacity of IRAK-M to inhibit TLR mediated cytokine production in human macrophages. Thus, we identified and characterized the primary interactive sites on the DD of IRAK-M on the basis of a structural model.

## Results

### Homology model of the human IRAK-M death domain

**Figure 1:**
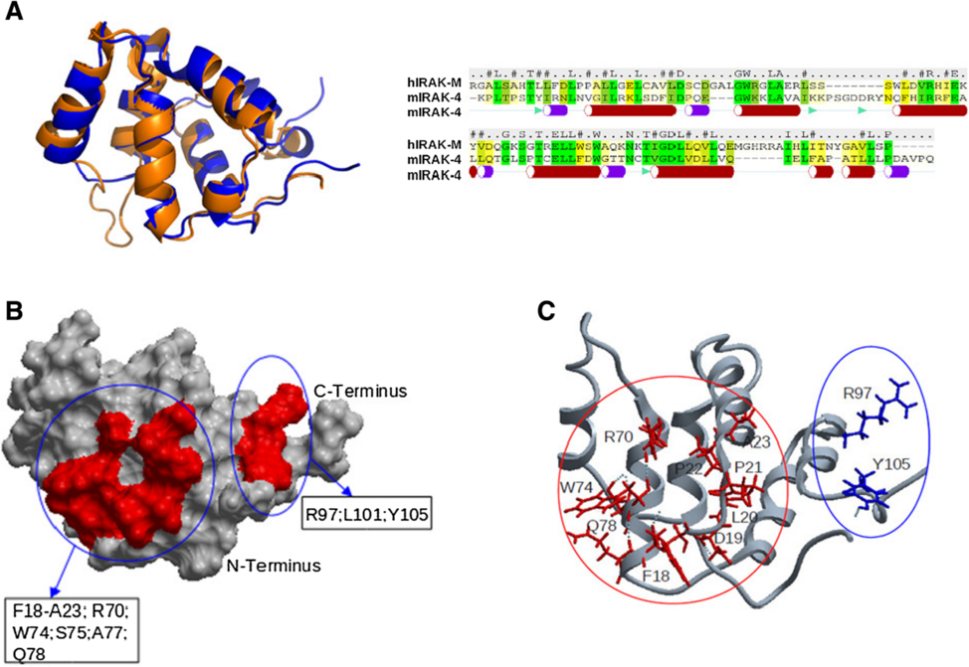
**3D structure model of the human IRAK-M death domain (DD). (A)** Model of the DD of human IRAK-M (Blue) superimposed on the template DD structure 2A9I (Orange) of mIRAK-4 and sequence alignment of hIRAK-M-DD and mIRAK-4-DD. The sequence identity is 28.7%. Secondary structures such as alpha-helices and beta-strands of mIRAK-4-DD (2A9I) are indicated underneath the sequences (Red bar: alpha-helix: Purple bar: unstable helix; Green arrow: beta-strand). **(B)** Interactive surface prediction of hIRAK-M-DD. Space filling model with predicted interactive residues in red that form two patches. **(C)** The predicted interactive residues in the two patches which were mutated in this study are shown with side chain and residue number in the back bone model in red and blue.

We generated a model for the death domain (DD) of human IRAK-M (IRAK-M-DD) by homology modeling based on the crystal structure of the DD of mouse IRAK-4 (PDB 2A9I [21], which has 28.7% sequence identity to the human IRAK-M DD as described in the Methods section). The generated IRAK-M-DD structure (Figure 1A) with 6 helical bundles forms a hydrophobic core that is decorated with a charged outer layer. An anti-parallel beta sheet, not seen in the template structure is formed by one strand from the N-terminus and another strand N-terminal of helix 5. An anti-parallel sheet located in between helix2 and helix3 of the template structure is absent in the DD of IRAK-M, instead a beta turn is made here by two serine residues in our model. Unconstrained molecular dynamics simulation for 100 nanoseconds (ns) indicated good stability of this structure (Additional file 1: Figure S1) and the quality of the structure was further verified by means of the total energy, root mean square deviation (RMSD) and the number of hydrogen bonds in the DD domain. Residues predicted to be involved in protein-protein interactions were identified as described in the Methods section. The identified potential interactive residues are present in areas formed by the N-terminus of helix1, the C-terminus of helix4, the loop between helix4 and helix5, and helix6 (Figure 1B and C).

### Functional analysis of DD mutagenesis after transient overexpression of IRAK-M variants in 293T cells

**Figure 2:**
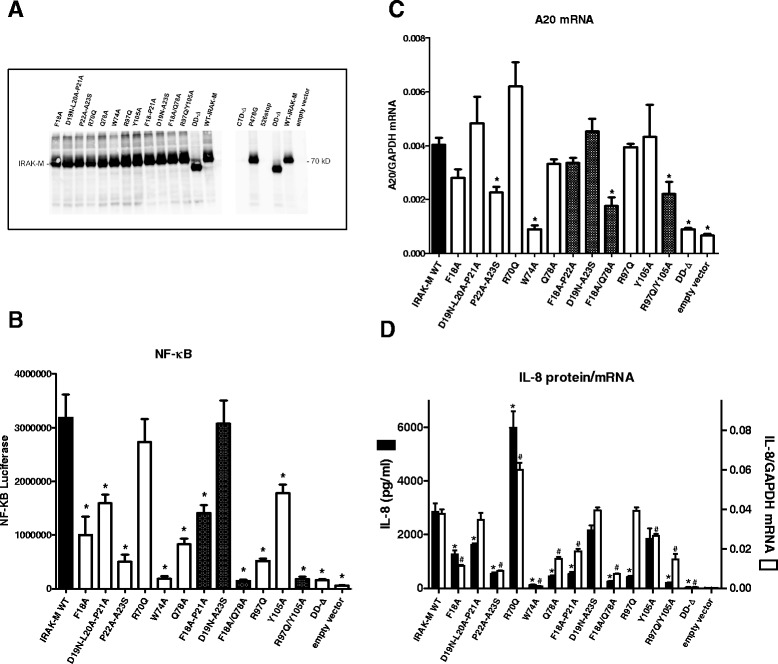
**Expression and functional analysis of human IRAK-M-DD mutants in 293T cells. (A)** Analysis of expression levels of IRAK-M variants by transfection in 293T cells by Western blotting performed on cell lysates with an antibody directed to the C-terminal of IRAK-M. **(B)** Effect of IRAK-M-DD mutations on NF-κB activation by overexpression in 293T cells. **(C)** Effect of IRAK-M-DD mutations on A20 mRNA induction by overexpression in 293T cells. **(D)** Effect of IRAK-M-DD mutations on IL-8 protein and mRNA production by overexpression in 293T cells. **(B-D)** N=4, mean±SEM. *Indicates difference with WT IRAK-M P<0.05. Shaded bars depict results of IRAK-M molecules with combinations of mutated residues/stretches within the same patch.

Based on our IRAK-M-DD model we probed potential interactive residues via structurally conservative mutagenesis of full-length human IRAK-M (Additional file 2: Table S1). The selected residues for mutation we predicted to be surface exposed and do not form intra-molecular contacts (see Figure 1C). In the binding area formed by helix1/helix4+loop we constructed a F18A mutant, generated a combined D19N-L20A-P21A mutant and a combined P22A-A23S mutant, and R70Q, W74A and Q78A single mutant molecules. We verified that all mutants were expressed in 293T cells at comparable levels by evaluation of expression levels with an antibody against the C-terminus of IRAK-M (Figure 2A). Overexpression of IRAK-M in 293T cells results in endogenous IRAK signaling. No major alterations were observed in the levels of the endogenous IRAK signaling pathway upon transfection of IRAK-M mutants (Additional file 1: Figure S2). We next determined the capacity of these human IRAK-M variants to activate NF-κB spontaneously when overexpressed in 293T cells as had been described for WT IRAK-M [1]. As shown in Figure 2B, human WT IRAK-M induces NF-κB in 293T cells, this function is fully dependent on the death domain since deletion of the death domain reduced this capacity to control level. Mutation of only F18 or D19-P21 only moderately affected the capacity of IRAK-M to induce NF-κB activity. Mutation of P22-A23 and Q78 caused a marked reduction in NF-κB. Single mutation of W74 completely abolished the capacity of IRAK-M to induce NF-κB in agreement with the study of Zhou *et al.* [13] of murine IRAK-M. Combined mutation of F18/D19-P21 did not result in a further reduction of NF-κB compared to the single mutants (Figure 2B). Notably, the combined mutation of D19-P21/P22-A23 resulted in a mutant that regained its full NF-κB activating capacity as compared to the P22-A23 mutant, which indicates that the D19-A23 stretch may harbor a negative control element. R70 mutation did not affect NF-κB activity. Combined mutation of F18/Q78, adjacent residues in the 3D structure, resulted in a mutant that fully lost its NF-κB activating capacity (Figure 2B). Thus it seems that W74 and F18/Q78 interactions are pivotal in the spontaneous NF-κB activating capacity of IRAK-M in 293T cells and that the D19-A23 stretch has only a regulatory role in this.

Mutation of R97 and Y105 in the interactive area of helix6, revealed that also R97 is of major importance in the NF-κB activating activity of IRAK-M in 293T cells while single mutation at Y105 exerts only a modest effect on NF-κB (Figure 2B). However, combined R97/Y105 mutation reduced NF-κB to the level of the death domain deletion mutant which identifies these residues as a second crucial binding site for the NF-κB activating activity of IRAK-M in 293T cells (Figure 2B).

IRAK-M has been reported to induce transcription of genes that do not rely on transcript stabilization such as the anti-inflammatory mediator A20 [13]. A20 transcription was induced by IRAK-M in 293T cells in a manner that appears primarily dependent on W74 (Figure 2C). Although R97 was involved in over 80% of the induced NF-κB (Figure 2B), the R97Q mutant generated the full A20 transcript potential. Importantly, while single mutation of R97 and Y105 did not impact on A20 transcript levels the R97Q/Y105A double mutant displayed significantly reduced levels of A20 mRNA, indicating a partial role of these helix 6 residues in A20 transcript expression.

Overexpression of IRAK-M in 293T cells also induced IL-8 secretion which involves NF-κB dependent transcription and translation [22]. The IL-8 production induced by the different DD-mutants in 293T cells (Figure 2D) occurred analogous to the NF-κB activity (Figure 2B). In line with the reduced NF-κB and IL-8 protein production capacity of the helix 1/4-5 mutants W74A, P22A-A23S and F18A/Q78A, the IL-8 transcript levels induced by these mutants were markedly reduced indicating pivotal involvement of these adjacent residues in transcription. In contrast IL-8 transcript production was not affected by mutation of R97, while IL-8 protein production by the R97Q mutant was 85% reduced (Figure 2D) indicating a role for this site in helix 6 in translation by a different pathway. Combined mutation of R97 and Y105 resulted in decreased IL-8 transcript levels (Figure 2D), but the relative impact on IL-8 protein production by the R97Q/Y105A mutation was importantly larger than that of the helix 1 mutants F18A and P22A-A23S with similar transcript levels (Figure 2D). The latter also points to involvement of the binding site in helix 6 of IRAK-M in protein translation. Remarkably, the R70Q mutant displayed significant hyperactivity with regard to IL-8 transcription and translation compared to WT IRAK-M. The combined D19-P21/P22-A23 mutant induced IL-8 protein and transcript levels equally well as WT IRAK-M.

### Functional analysis of C-terminal domain mutants after transient overexpression of IRAK-M variants in 293T cells

**Figure 3:**
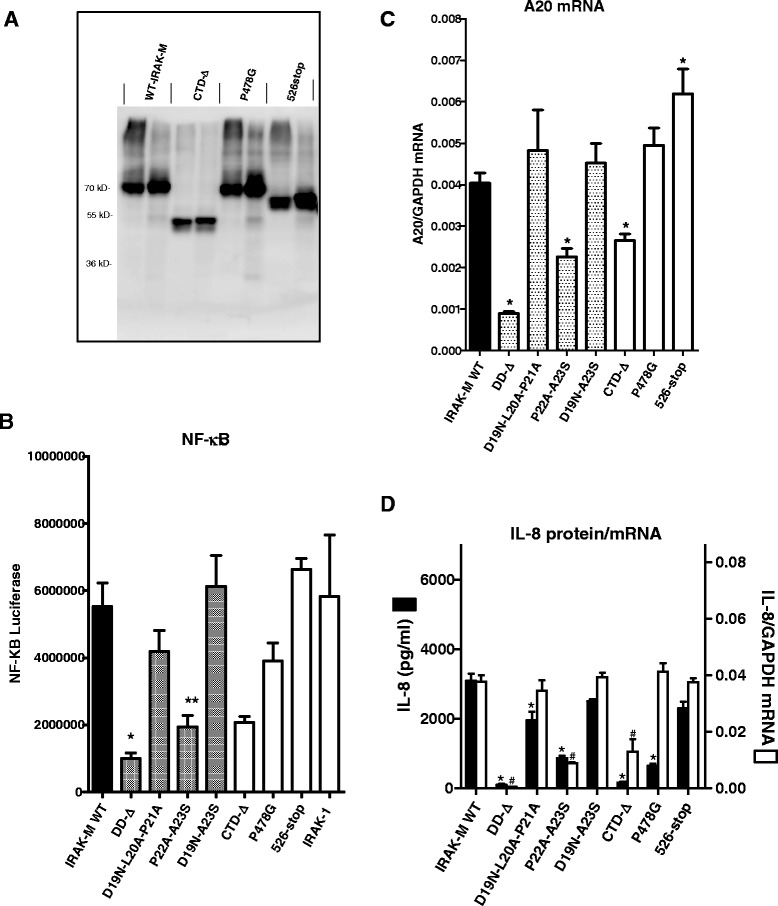
**Expression and functional analysis of IRAK-M C-terminal domain mutants in 293T cells. (A)** Analysis of transient expression of IRAK-M and C-terminal domain mutants by transfection in 293T cells by Western blotting performed on cell lysates with an antibody directed to full length IRAK-M. **(B)** Effect of IRAK-M C-terminal domain mutation on NF-κB activation by overexpression in 293T cells. **(C)** Effect of IRAK-M C-terminal domain mutation on A20 mRNA induction by overexpressing in 293T cells. **(D)** Effect of IRAK-M C-terminal domain mutation on IL-8 protein and mRNA production by overexpression in 293T cells. (B-D) Comparison to IRAK-M-DD mutants in the D19-A23 stretch. N=4, mean±SEM. *Indicates difference with WT IRAK-M P<0.05.

IRAK-M dependent NF-κB activation proceeds in IRAK-1/IRAK2 double deficient cells through a unique MEKK3 pathway [13]. Taking this into account it can be hypothesized that IRAK-4/IRAK-M complexes recruit MEKK3 and activate TRAF6 in a specific manner that is dependent on a putative TRAF6 binding motif in the IRAK-M C-terminal domain (CTD) that extends from the inactive kinase domain. Because no experimental data existed yet on the functional involvement of the CTD (amino acid S445-E596) of IRAK-M, we generated an IRAK-M variant truncated at position S445 that lacks the entire CTD (CTD-∆). Furthermore we mutated the P^478^VEDDE^483^ TRAF6 binding motif [10] in the CTD by introduction of a P478G substitution and generated a mutant that lacks the C-terminal part of the CTD by truncation at position K526. The P478G mutant was expressed similar as WT IRAK-M, and, as anticipated, the CTD deletion and K526 truncation mutant were not recognized by antibodies directed against the C-terminus of IRAK-M (Figure 2A). A polyclonal antibody raised against full-length IRAK-M displayed the expression of these CTD mutants (Figure 3A). Deletion of the complete CTD resulted in a major reduction of the NF-κB activating capacity of IRAK-M when overexpressed in 293T cells, while the P478G mutation in the TRAF6 binding motif led to a non-significant reduction in NF-κB (Figure 3B). Truncation of the CTD at position K526 did not affect NF-κB activity. Together these data indicate a major involvement of the CTD in the NF-κB activating capacity of IRAK-M, which appears independent of the TRAF6 binding motif at P478.

Deletion of the CTD of IRAK-M resulted in a partial reduction of the A20 mRNA levels induced by IRAK-M. The TRAF6 binding site at P478 was not involved in the expression of A20 transcripts (Figure 3C). Remarkably, truncation of the IRAK-M CTD at K526 somewhat potentiated A20 transcript levels in 293T cells.

The reduced capacity of the IRAK-M CTD-∆ mutant to activate NF-κB was associated with an almost complete loss of IL-8 production (Figure 3D). Interestingly, the TRAF6 binding motif at P478 appeared essential for IL-8 production by IRAK-M, this while NF-κB was hardly affected by the P478G substitution (Figure 3B and D). For comparison the DD-mutants D19-A21 and P22A-A23S mutant were studied simultaneously. These mutants displayed NF-κB levels comparable to the P478G mutant and CTD-∆ mutant, however the CTD mutants displayed a much larger effect on IL-8 protein production compared to these DD-mutants. NF-κB, IL-8 transcription and translation correlated well for the D19-A21 and P22A-A23S mutants. The TRAF6 binding site mutant P478G however induced IL-8 transcripts as efficient as WT IRAK-M, while IL-8 protein levels induced by this mutant are 80% reduced (Figure 3D) which indicates involvement of the TRAF6 binding site in IL-8 translation upon transfection of IRAK-M in 293T cells. IL-8 transcription was induced by the CTD-∆ mutant (Figure 3D) albeit at a lower level than was observed for WT IRAK-M, which may indicate a role for the CTD in IL-8 transcription or mRNA stabilization in 293T cells. Truncation of the CTD of IRAK-M at position 526 did not significantly affect IL-8 transcript levels or protein expression.

### Modeling of putative IRAK-M-DD tetramer and protein docking in the myddosome structure

**Figure 4:**
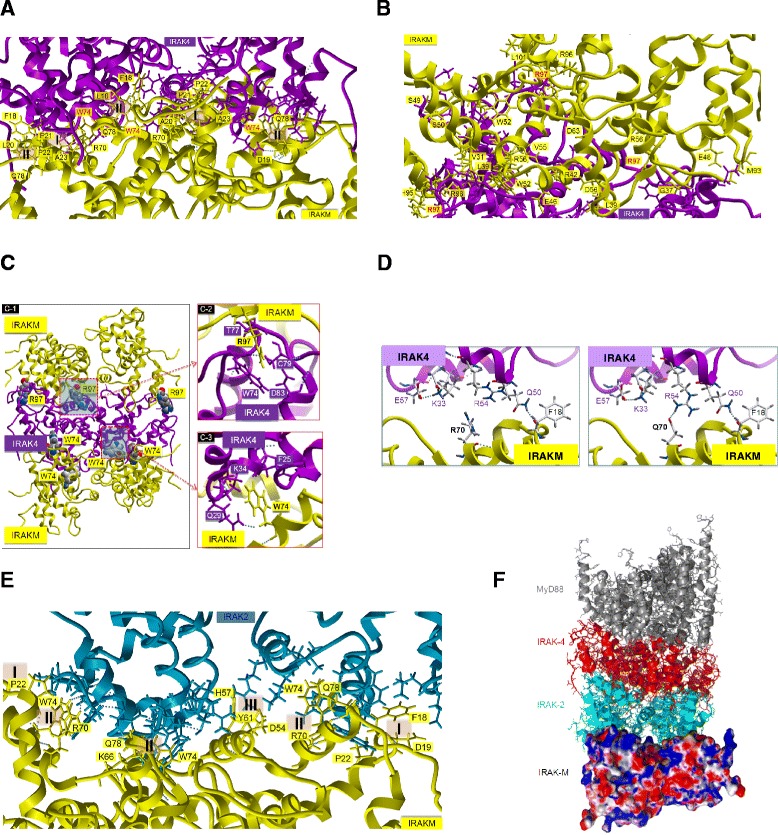
**Protein:protein docking of IRAK-M-DD tetramers onto IRAK-4-DD tetramers or IRAK-2-DD tetramers. (A)** IRAK-M-DD interactions predicted to be important for the contact between IRAK-M and IRAK-4 as obtained by unbiased docking of the IRAK-M tetramer (Yellow) onto the *bottom* surface of the IRAK-4 tetramer structure (Purple) as is present in the myddosome (3MOP.pdb also **F**). The residues involved in the interaction are shown with their side chains, of which the residues from IRAK-M are labeled with their respective residue name and number. Two interaction types were involved (type I residues L20, P21, P22, A23, R70, type II residues L16, F18, W74, S75, Q78). This orientation was observed for 63% of the 100 best docked events. **(B)** IRAK-M-DD interactions predicted to be important for the contact between IRAK-M and IRAK-4 as obtained by unbiased docking of the IRAK-M tetramer (Yellow) on the top surface of IRAK-4 tetramer (Purple). The residues involved in the interaction are shown with their side chains, including R97. This orientation was observed for 37% of the 100 best docked events. **(C)** Potential sandwich of one IRAK-4-DD tetramer in between 2 IRAK-M-DD tetramers by W74 and R97 mediated interactions. The respective predicted IRAK-4 interactions are shown in detail. **(D)** Docking of mutant IRAK-M-DD predicts increased IRAK-4 interaction with the IRAK-M R70Q mutant through an extra hydrogen bond formed between Q70 in IRAK-M and R54 in IRAK-4. **(E)** Part of the composite binding site of the free side of the IRAK-2-DD tetramer in the myddosome is shown with three IRAK-M-DD molecules. The contact residues are shown with their side chain. The residues involved in the interaction from IRAK-M are labeled. Three interaction types were involved. **F)** A proposed whole myddosome structure with IRAK-M-DD tetramer docked on the free side of the IRAK-2-DD tetramer, based on the myddosome structure, 3MOP.pdb.

Our structural model for the DD of IRAK-M appeared a reliable tool to guide our mutagenesis experiments. This prompted us to use the model in protein docking simulation experiments. The DDs of IRAK-4 and IRAK-2 form homotetramers in the myddosome structure [3] and we could superimpose four of our IRAK-M-DD model structures in either the coordinates of the IRAK-4 or IRAK-2 tetramer of the reported myddosome structure (3MOP, [3]) as depicted in Additional file 1: Figure S3A. Figure S3B in Additional file 1 shows the predicted IRAK-M-DD interactions involved in the formation of IRAK-M-DD homotetramers which include F18, P22, A23, L25, G65 and R70 of one DD and L53, K60 and Q64 of another IRAK-M-DD. The observation that both W74 and R97 are pivotal for the IRAK-M activity towards NF-κB and the notion that IRAK-M mediated NF-κB is IRAK-4 dependent [13] prompted us to model the interaction of the IRAK-M-DD tetramer structure with the IRAK-4-DD tetramer derived from the earlier experimentally determined myddosome structure, deposited as the 3MOP.pdb structure. In the 3MOP structure, a tetramer of IRAK-4-DD’s interacts with a tetramer of the DD’s of MyD88 on one side, and a tetramer of IRAK-2-DD’s on the other side. Unbiased *in silico* protein docking of the IRAK-M-DD tetramer onto the IRAK-4-DD tetramer side which interacts with IRAK-2 in 3MOP displays a W74 dependent interaction of IRAK-M with IRAK-4 (Figure 4A) in accordance with the reported W74 importance for IRAK-4 binding [13]. However no contact point is predicted for R97 in this type of interaction given that R97 is in fact located at the opposite side of the W74 interacting tetramer surface. However when the R97 exposing side of the IRAK-M-DD tetramer was docked unbiased at the top side of the IRAK-4 tetramer (Figure 4B) there were significant contacts noted with R97 interacting with IRAK-4 residues W74, T77, C79 and D83 (Figure 4C2). When IRAK-M tetramers were docked onto IRAK-4 tetramers completely without any restrictions, we observed two different overall docking poses: 63% of the binding events were with the W74 exposing side and 37% with the R97 side. Our results indicate that IRAK-M may actually have the capacity to bind, oligomerize and activate IRAK-4 in a MyD88 type of manner to induce NF-κB, transcription and translation via respectively IRAK-1 and IRAK-2. Furthermore, there is also the possibility that one IRAK-4 tetramer may actually form a complex with two IRAK-M tetramers on either side as depicted in Figure 4C1.

We observed that the IRAK-M-DD R70Q variant displays hyper-reactivity towards IL-8 production when expressed in 293T cells (Figure 2D). Structure analysis of the DD-model indicated that the R70Q variant may actually form an extra hydrogen bond with R54 of IRAK-4 (Figure 4D). Remarkably, the extra interaction with IRAK-4 of R70Q led to increased transcript and protein levels of IL-8 without an increase in total NF-κB (Figure 2B,D).

Unbiased docking of the IRAK-M-DD tetramer to the free side of the IRAK-2-DD tetramer in 3MOP (see Figure 4F) indicated the W74 side to be plausibly involved in protein-protein contacts (Figure 4E) without involvement of R97. R70 is predicted to be involved in the interaction with IRAK-2 (Figure 4E), but different from the interaction gain with IRAK-4, the R70Q mutant is predicted to lose the IRAK-2 interaction points at position 70 of IRAK-M.

### Potency of IRAK-M-DD mutants to inhibit TLR signaling in macrophages

**Figure 5:**
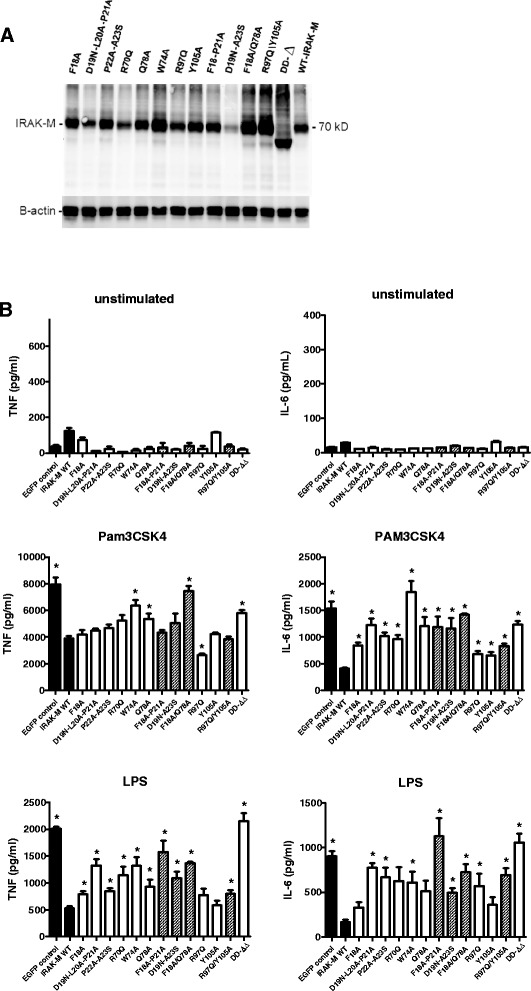
**Effect of human IRAK-M and death domain mutants in macrophages.** WT IRAK-M and DD-mutants were stably expressed in the human monocytic cell line THP-1 by lentiviral transduction and FACS-sorting of EGFP positive cells which is bicistronically expressed. THP-1 cells were matured to macrophages before stimulation as described in Methods. **(A)** IRAK-M expression evaluated by western blotting. **(B)** The effect of stable WT IRAK-M expression and IRAK-M mutants was determined on TLR2 (Pam3CSK4) and TLR4 (LPS) mediated TNF and IL-6 production after stimulation for 6 hour. Shaded bars depict results of IRAK-M molecules with combinations of mutated residues/stretches within the same area. N=4, mean±SEM. *Indicates a difference with WT IRAK-M P<0.05.

**Figure 6:**
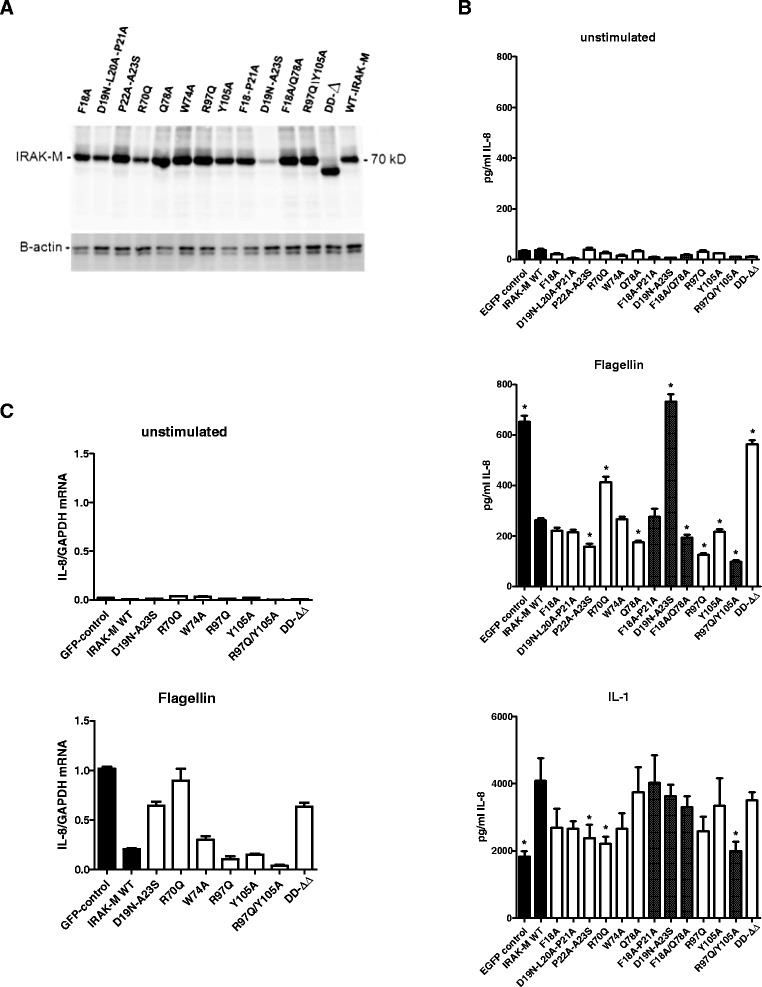
**Effect of human IRAK-M and death domain mutants in lung epithelial cells.** WT IRAK-M and DD-mutants were stably expressed in the lung epithelial cell line H292 by lentiviral transduction and FACS-sorting of EGFP positive cells which is bicistronically expressed. **(A)** IRAK-M expression evaluated by western blotting. **(B)** The effect of stable IRAK-M expression and IRAK-M mutants was determined on IL-1β and Flagellin (TLR5) mediated IL-8 expression in the supernatant after 6 hour stimulation. Shaded bars depict results of IRAK-M molecules with combinations of mutated residues/stretches within the same area. N=8, mean±SEM. * indicates difference with WT IRAK-M P<0.05. **(C)** Effect of stable expression of WT and DD domain mutants on IL-8 mRNA levels induced by Flagellin after 3 hours. N=3, mean±SEM.

IRAK-M is expressed in monocytes/macrophages and in lung epithelial cells and IRAK-M is an important factor to down-regulate host defense in bacterial pneumonia models [1,14-16,19]. In order to study the potential pro- and anti-inflammatory effects of human WT IRAK-M and the DD mutants on TLR mediated cytokine/chemokine release we stably introduced these proteins in human monocytic cells (THP-1) and in human bronchial lung epithelial cells (H292). Coding sequences of WT and mutant IRAK-M were stably introduced by a lentiviral system with bicistronic expression of eGFP that enabled FACS-sorting of eGFP positive cells such as to obtain IRAK-M expressing cells of identical and homogeneous transcription for WT and mutant transgenes. Most mutants showed comparable or somewhat higher protein levels than WT IRAK-M upon stable expression in THP-1 and H292 cells (Figures 5A and 6A), however the hyperactive mutant R70Q displayed lower expression, and the D19N-A23S mutant displayed markedly lower steady state protein levels. Identical levels of IRAK-M mRNA were observed for the R70Q and D19N-A23S mutants as for WT IRAK-M (Additional file 1: Figure S4A), and since all mutants showed similar protein expression upon transient expression in 293T cells (Figure 2A) it appears that the R70Q and D19N-A23S mutants are prone to increased protein turnover in a more proficient cell type. Inhibition studies with proteasome inhibitor MG-132 or an IRAK-1/4 inhibitor did not normalize expression of these mutants. Thus the mechanism underlying the high turnover and/or low protein expression level of these mutants remains elusive, although it may correlate with their relatively high activity.

Overexpression of WT IRAK-M in human THP-1 derived macrophages significantly inhibited TLR2 and TLR4 induced TNF and IL-6 production in a death domain dependent manner (Figure 5B). Mutation of the residues D19-P21, P22-A23, W74 and Q78 resulted in partially or completely restored TNF and IL-6 production in macrophages (Figure 5B). In contrast, single or combined mutation of R97 and Y105 in helix 6 did not or hardly influence TNF production induced by respectively TLR2 or TLR4 when compared to WT IRAK-M (Figure 5B). Single R97Q mutation even resulted in increased capacity of IRAK-M to inhibit Pam3CSK4 induced TNF production, when compared to WT IRAK-M . Pam3CSK4 induced IL-6 release was however partially restored by R97 and Y105 mutation and more so in case of the R97Q/Y105A double mutant. Remarkably, mutation of R97 and combined R97/Y105 mutation reduced the capacity of IRAK-M to inhibit LPS induced IL-6 production considerably and the helix 6 interaction appeared equally important for inhibition of LPS induced IL-6 by IRAK-M as the D19-P21, P22-A23 and W74 type of interactions (Figure 5B). Although the steady state expression level of the D19-A23 mutant is low in macrophages (Figure 5A) this mutant still reduced LPS mediated TNF and IL-6 production significantly compared to the eGFP-control (Figure 5B). These results indicate that IRAK-M inhibits cytokine production in macrophages with a dominant role for W74 and surrounding residues in controlling TNF production, and a differential role for the helix 6 residues R97 and Y105 which appear to control LPS mediated IL-6 by IRAK-M in cooperation with residues on the other side of the putative IRAK-M tetramer.

### Potency of IRAK-M-DD mutants to inhibit TLR5 and IL-1β signaling in lung epithelial cells

IRAK-M has been reported to be expressed in lung epithelial cells [19]. Lung epithelial cells are relatively unresponsive to TLR2 and TLR4 agonist, but react potently to MyD88/IRAK dependent IL-1 receptor and TLR5 stimulation [23], which we could confirm with the lung epithelial cell line H292. The most prominent inflammatory mediator produced by these cells is IL-8. TLR5 is activated by Flagellin derived from the flagella of certain Gram-negative bacteria such as *P. aeruginosa.* In this respect it is noteworthy that IRAK-M^−/−^ mice are protected from *P. aeruginosa* pneumonia under immunocompromised conditions caused by cecal ligation and puncture [14]. TLR5 mediated IL-8 production by H292 lung epithelial cells after Flagellin stimulation was significantly inhibited by WT IRAK-M (Figure 6B). The inhibition of TLR5 mediated IL-8 production by IRAK-M was completely death domain dependent since the DD-deletion mutant showed no effect on IL-8 production. The R70Q mutation was associated with partially restored IL-8 production. Mutagenesis of the D19-A23 stretch resulted in complete restoration of chemokine production. Interestingly mutation of key residue W74 did not influence the inhibitory effect exerted by IRAK-M on TLR5 mediated IL-8 production. Remarkably R97 and Y105 mutagenesis prominently enforced the inhibitory capacity of IRAK-M to inhibit TLR5 mediated IL-8 production (Figure 6B), an effect that was also observed for some other mutants (P22A-A23S and Q78A). The inhibitory action of IRAK-M and mutants on Flagellin induced IL-8 release was in line with the level of induced IL-8 transcripts indicating an inhibitory control in this setting by IRAK-M on IL-8 transcription and/or transcript stabilization (Figure 6C).

In contrast to the inhibition of TLR5 stimulated IL-8 expression, the IL-1β stimulated IL-8 expression by H292 lung epithelial cells was enhanced by WT IRAK-M (Figure 6B). The latter is however consistent with the notion that IRAK-M may substitute for IRAK-1 in IL-1β signaling [1]. Most DD-mutants did not show this stimulatory effect, interestingly however the DD-deletion mutant which lacks the entire DD also displayed some unexplained stimulation of IL-1β driven IL-8 expression.

To evaluate whether the dampening of TLR5 mediated IL-8 production by IRAK-M was caused by increased mRNA expression of negative feedback inhibitors such as A20 [13] we determined A20, SHIP-1 and SOCS-3 transcript levels in H292 cells that overexpressed WT or mutant IRAK-M proteins after 3 hours of stimulation. Instead of inducing A20 levels, IRAK-M appeared to inhibit Flagellin induced A20 and SOCS-3 expression in H292 cells (Additional file 1: Figure S5), and while IRAK-M seemed to up regulate basal SHIP-1 levels, this effect however appeared to be retained by the DD-deletion mutant that did not inhibit IL-8 release. Thus, our results suggest that IRAK-M regulates TLR5 mediated responses in lung epithelial cells by inhibition of the forward signaling to IL-8 induction, not by increase of negative feedback inhibitors. Remarkably however Flagellin-induced A20 expression appeared importantly higher in the case of the W74A mutant compared to WT IRAK-M, while IL-1β induced A20 expression appeared reduced for the R97 and R97/Y105 mutants (Additional file 1: Figure S5).

## Discussion

**Figure 7:**
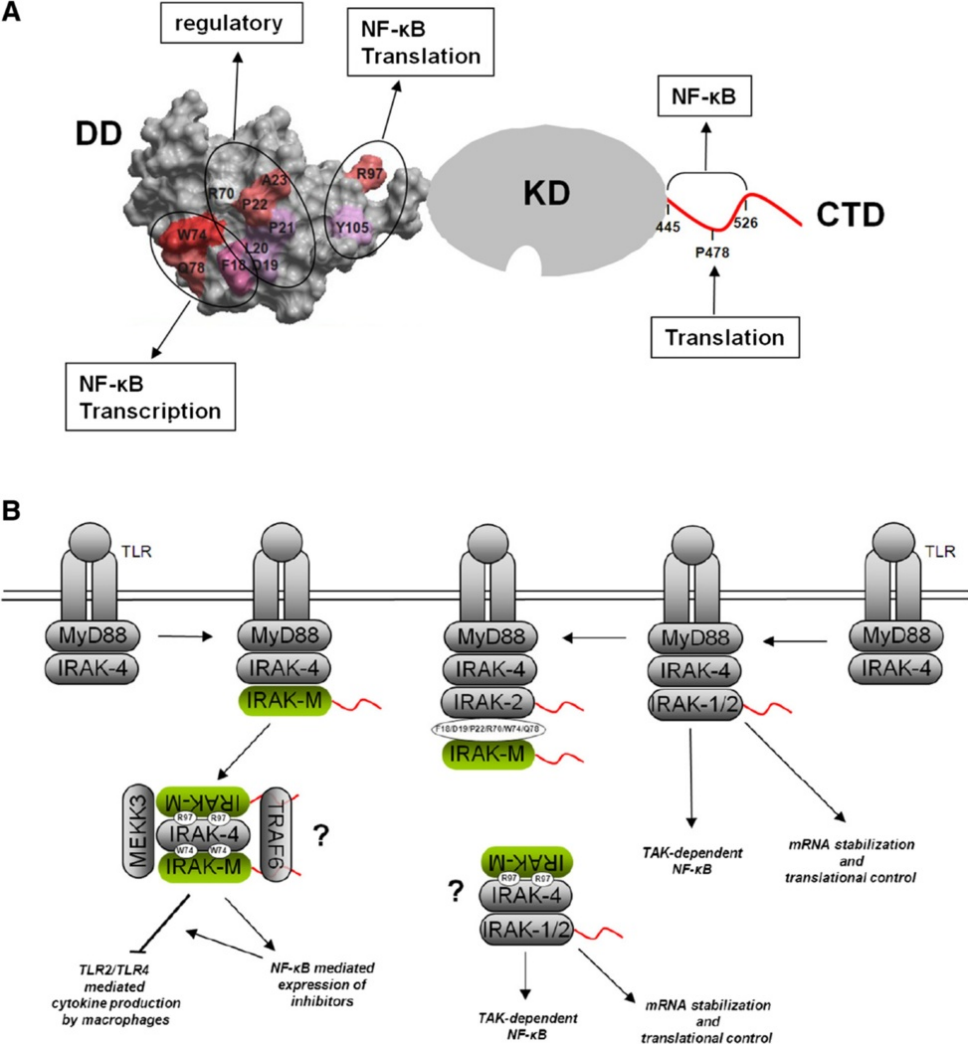
**Schematic representation of the structure-function relationships of IRAK-M based on this study. (A)** Functional involvement of the different residues of the Death Domain (DD) of IRAK-M on induction of NF-κB, transcription and translation upon overexpression in 293T cells (data derived from Figures 2 and 3). Graded color code (red->pink->white) indicates the impact of the single mutants on NF-κB in 293T cells (from red important to white no effect). The stretch D19-A23 is not indicated to be essential to NF-κB because mutation of the complete stretch (combined D19-P21 and P22-A23 mutation) results in a mutant with normal NF-κB. **(B)** Schematic representation of the working mechanism of IRAK-M as inhibitor of MyD88 signaling adapted from Zhou *et al.* [13], in which IRAK-4/IRAK-M interaction induces transcription of other inhibitors via MEKK3 dependent NF-κB and in which IRAK-2 mediated mRNA stability and translation is inhibited by IRAK-M. Based on our mutagenesis studies both W74 as well as R97 in the IRAK-M-DD are pivotal for IRAK-4/IRAK-M mediated NF-κB activity, but R97 is predicted to bind on the other side of IRAK-4 (see Figure 4). This may induce oligomerization of IRAK-4 and IRAK-1 or IRAK-2 activation, or, since both residues are essential for the unique, previously shown MEKK3 dependent IRAK-M mediated NF-κB activation, IRAK-4 may be sandwiched by IRAK-M to come to this pathway as indicated by W74 and R97 interactions. R97 is not predicted to be involved in the interaction of IRAK-M with IRAK-2 for which F18, D19, P22, R70 and W74 are predicted to be involved.

This report represents the first detailed structure function analysis of the death domain (DD) of human IRAK-M. Despite the fact that IRAK-M acts as an inhibitor of inflammation, IRAK-M paradoxically stimulates both NF-κB and transcription of inflammatory mediators [1,13]. Part of the anti-inflammatory effect of IRAK-M lies in the capacity of the molecule to deviate the pro-inflammatory NF-κB signals produced by IRAK-4 via IRAK-1 and IRAK-2 to a more anti-inflammatory IRAK-4/IRAK-M/MEKK3 NF-κB signal [13]. Zhou and coworkers convincingly displayed this IRAK-M transmitted NF-κB activity in the murine system using IRAK-1/IRAK-2 double deficient cells and IRAK-1/IRAK-2/IRAK-M triple deficient cells derived from knockout mice [13]. In the human system IRAK-1/IRAK-2 double deficient cells are not available and we used the well-described capacity [1] of IRAK-M to drive NF-κB activation spontaneously when transfected in 293T cells as a model system to study the NF-κB activating activity of human IRAK-M in cells of human origin. By mutation of predicted interactive sites, identified by homology modeling techniques, we demonstrated the involvement of at least 3 areas on the IRAK-M DD in the endogenous capacity of IRAK-M to spontaneously activate NF-κB, and to induce A20 transcription and IL-8 expression upon overexpression in 293T cells (Figure 2). A synopsis of our results is depicted in Figure 7A with a spacefilling model of the IRAK-M DD attached to a schematic KD and CTD. Consistent with the findings of Zhou *et al.* [13] with murine IRAK-M we observed that W74 is pivotal for the NF-κB activating activity by IRAK-M, as well as the surrounding F18 and Q78. In addition we found that R97 and Y105, located on the other side of the DD, can be of major importance for a large part of the NF-κB activating activity of IRAK-M. In Figure 7A the importance of the DD residues in the endogenous NF-κB activating activity of IRAK-M is indicated from red (important) to white (not important) based on the functional analysis of mutants which involved alterations of only 1 or 2–3 consecutive residues. In the predicted interactive area located between W74 and R97, both the D19N-L20A-P21A mutant as well as the P22A-A23S mutant had a partial effect on NF-κB expression. However, the combined D19-A23 mutant regained its full NF-κB activating activity, which indicates that the D19-A23 stretch is dispensable for NF-κB by IRAK-M and rather has a regulatory role. IL-8 transcription and protein expression induced by IRAK-M overexpression in 293T cells can be uncoupled by mutation of IRAK-M DD residue R97. This infers that the pathway activated by the R97 interface is completely different from the pathway activated by the W74 interface which is we found to be absolutely essential to both IL-8 transcription and expression in this 293T model. Consistent with the latter observation, protein docking experiments suggested that R97 is located on the opposite side of the putative IRAK-M tetramer, at a site that is predicted to bind only to the side of the IRAK-4 tetramer that binds to MyD88 in an experimentally determined myddosome structure (Figure 4). Interaction to IRAK-2 tetramers was predicted only for the binding interface involving W74 of the IRAK-M-DD tetramer and the free side of IRAK-2 tetramer in the myddosome. The potential interactions in the cell of IRAK-M with IRAK-4 and IRAK-2 based on these predictions are schematically depicted in Figure 7B.

It should be mentioned that we modeled the interactions of IRAK-M with other IRAK family members as if IRAK-M forms homotetramers and interacts as such with homotetramers of IRAK-4 and IRAK-2. This is based on the experimentally verified homotetramer formation observed for the isolated death domains of MyD88/IRAK-4/IRAK-2 when co-crystallized [3]. Full-length proteins may however interact differently, for instance the intermediate domain that extends immediately from the DD’s of MyD88 and IRAK-1 are extremely important for function [24,25]. Also in this regard we show here that the C-terminal domain of IRAK-M is functional and contains an active TRAF6 binding site (Figure 3).

With respect to structure-based predictions the results with the R70Q IRAK-M mutant are of major importance. As may be inferred from protein docking experiments, the R70Q mutant tetramers may form an extra hydrogen bond with R54 of IRAK-4 tetramers, but loose the R70 interaction points with IRAK-2. In line with its increased IRAK-4 interaction, the R70Q mutant displayed hyperactivity towards IL-8 transcription compared to WT when overexpressed in 293T cells. IL-8 transcription in this *in vitro* setting is primarily W74 driven and indeed the IRAK-4 interaction predicted to be enforced by the R70Q variant involves the W74 interface. Also IL-8 protein expression was increased in 293T in case of the R70Q mutant. Translation induced by IRAK-M in 293T cells appears primarily R97 driven, presumably through a MyD88 type of interaction of IRAK-M with IRAK-4, which may drive IRAK-2 activity to translation of inflammatory genes [13] as depicted in Figure 7B. However, this potential IRAK-2 translational activity induced by IRAK-M will be, as in the myddosome, also open for inhibition by IRAK-M. The predicted loss of an interaction site of the IRAK-M R70Q mutant with IRAK-2 will relieve this IRAK-2 inhibition which contributes to the observed increased IL-8 protein expression. The observations of a “gain of function” mutant in this regard confirm the quality and predictive power of our IRAK-M-DD model.

To display an altered anti-inflammatory capacity of the IRAK-M DD mutants we introduced them in THP-1 macrophages and H292 lung epithelial cells. Zhou *et al.* [13] showed that W74 is involved in IRAK-4 binding and NF-κB activating activity of IRAK-M when macrophages are stimulated with a viral type TLR7 agonist. Here we show that W74 is dominantly involved in the capacity of IRAK-M to reduce TLR2 and TLR4 mediated cytokine release in macrophages. To our knowledge this is the first demonstration that W74 is involved in the inhibitory actions of IRAK-M. Also surrounding residues of W74 (F18/Q78, D19-P21, P22-A23, R70) displayed major contributions to the capacity of IRAK-M to inhibit TNF and IL-6 production by Pam3CSK4 and LPS in macrophages. However, the D19-A23 mutant remained able to reduce LPS induced TNF and IL-6 production, which is in line with the dominant role of W74 in this feature and an active mode of action of IRAK-M as proposed by Zhou *et al.* [13]. Mutation of R97 and Y105 in helix 6 had little effect on the capacity of IRAK-M to inhibit TLR2/4 stimulated TNF production in macrophages, but did exert a major drop in the potency of IRAK-M to inhibit LPS stimulated IL-6 production. Thus, the involvement of residues R97/Y105 of IRAK-M to inhibit cytokine produced in macrophages is dependent on the trigger and cytokine evaluated. Analogously, TNF and IL-6 expression are differentially regulated, with TNF being identified as a primary response gene [26]. The observation that W74 as well as R97 are involved in the inhibition TLR mediated of IL-6 production indicates an important role for the R97 type of interaction with IRAK-4 that could either compete for MyD88 binding to IRAK-4 or provide a special NF-κB signal to express anti-inflammatory inhibitors.

In contrast to the importance of residues W74 and R97 in aforementioned activities of IRAK-M these residues were not implicated in the inhibition by IRAK-M of TLR5 mediated IL-8 production by lung epithelial cells (Figure 6B). Since W74 of IRAK-M is pivotal for IRAK-4 interaction [13] it appears that the functionality of IRAK-M in the inhibition of TLR5 mediated IL-8 in these epithelial cells is independent of its interaction with IRAK-4. The R97 mutant displayed even a robust increased inhibitor potency on TLR5 mediated IL-8 production, which may be related to the reduced IRAK-4 binding possibilities of this mutant and therefore potentially increased IRAK-2 binding events. Interestingly increased expression of the inhibitor A20 by IRAK-M as reported [13] was not associated with the inhibitory effect of IRAK-M in the lung epithelial cell experiments presented here (Additional file 1: Figure S5). Taken together it appears that the inhibitory action of IRAK-M on TLR5 mediated IL-8 production in lung epithelial cells is mainly the result of inhibition of the functions of IRAK-2. In line, posttranscriptional processes are reported to dominate TLR5 mediated IL-8 production in epithelial cells [27] and transcript stabilization and translation are pathways induced via IRAK-2 [13].

The differential importance of the alternate DD sites of IRAK-M in their inhibitory functioning under varying conditions is probably due to distinct limiting factors in the involved pathways. Recent discoveries such as the cell specific regulation of IL-6 production by TLR dependent autophosphorylation of IRAK-4 [28] may turn out to be pivotal in our understanding of IRAK-M as an inhibitor of IRAK-4 dependent processes. It should be stressed that while we used a theoretical 3D model to rationalize the in vitro experiments, our results show the involvement of the different residues of IRAK-M at the functional level only. Additional studies are needed to confirm or refute the potential interactions predicted by our protein modeling studies.

A naturally occurring P22L IRAK-M mutant is reported to be associated with early onset asthma [19]. Residue P22 is predicted to be involved in IRAK-2/4 and M tetramer interactions (Figure 4 and Additional file 1: Figure S3b). The enhanced propensity of individuals with the P22L mutation to develop asthma is in line with the observed decreased capacity of IRAK-M with mutations in this region (P22A-A23S mutant) to down-regulate IL-6 expression in macrophages upon TLR2/4 stimulation (Figure 5B).

From the overexpression experiment in 293T cells it could be concluded that residues D19-A21 and P22-A23 are involved in both positive and negative effects on signaling events. This is consistent with our finding that the D19-A21 as well as the P22-A23 mutant IRAK-M proteins lack capacity to inhibit cytokine expression in macrophages (Figure 5B). This would involve reduced IRAK-4 interaction of IRAK-M at L20, P21, P22 and A23 as well as reduced IRAK-2 interaction of IRAK-M at D19 and P22.

Interestingly, the relative high NF-κB activity of the D19-A23 IRAK-M mutant (Figures 2B and 3B) is associated with low protein levels of this mutant in proficient cells (Figures 5A and 6A). For IRAK-1 it has been suggested that an intramolecular interaction of the death domain with the end of its CTD keeps IRAK-1 in a silent mode before phosphorylation events trigger its activation [29]. IRAK-1 that lacks the end of its CTD is easily activated and instable [28]. In the context of IRAK-M one could hypothesize a role of the D19-A23 stretch in the stability of IRAK-M either by interaction with its own CTD or specific interaction/repelling with/of other IRAK or associated molecules. The lower observed steady state levels of the IRAK-M mutant protein with the modified D19-A23 stretch (Figures 4A and 5A) would be consistent with faster turnover through increased kinetics of activation and subsequent degradation events. In this respect, blotting experiments with the IRAK-M K526stop mutant that lacks the C-terminal epitope recognized by the used C-terminal anti-IRAK-M antibody, showed clearly that both the low and high molecular bands observed upon expression of IRAK-M are specific IRAK-M derived products. These products appear to display the continuous modification and degradation of WT IRAK-M as well as the mutants (Figures 2A, 5A, 6A). The phenotype observed with the K526stop mutant was increased A20 transcription (Figure 3C) which could support the proposed presence of a regulatory element in the very C-terminal end of IRAK-M analogues to the CTD of IRAK-1 [28].

We compared the homologous residues involved in the function of IRAK-M with those of other IRAK family members (Additional file 1: Figure S6). The stretch F18-A23 in IRAK-M, which is involved in NF-κB activating activity as well as inhibitory activity (Figures 2, 3, 5 and 6), is least conserved among IRAK proteins (Additional file 1: Figure S6D). Major difference with IRAK-2 is that A23 in IRAK-M is a bulky and aromatic tryptophan at the homologous position in IRAK-2 (W11) (Additional file 1: Figure S6A and B). Furthermore the negative charge provided in IRAK-M by D19 is absent in IRAK-2 (Q7). In IRAK-4 this negative charge is also lacking at the homologous position (C13), instead a positive charge is provided in IRAK-4 in this area by R12 in the homologous position of the aromatic phenylalanine (F18) in IRAK-M (compare Additional file 1: Figure S6E1 and E3). Undoubtedly these differences will contribute to the divergent functions of the different homologous DD. The residues in the D19-A23 stretch are predicted IRAK-M interaction points with IRAK-M itself (P22, A23), with IRAK-4 (L20, P21, P22, A23), and IRAK-2 (D19, P22) (Figure 4).

A rare naturally occurring R97Q IRAK-M mutant has been reported (rs185025028). Here we show that the R97 binding interface can be of importance for the functioning of IRAK-M depending on the cell type, stimulus and readout. As mentioned, the R97Q mutation has a phenotype with regard to the functioning of IRAK-M in macrophages (lower potency to inhibit TLR4 elicited IL-6) and lung epithelial cells (higher potency to inhibit TLR5 elicited IL-8). It will be interesting to see whether the R97Q genotype is associated with altered disease phenotype or outcome. R97 plays an important role in the functioning of IRAK-M in cooperation with Y105. R97 is conserved in IRAK-2, but not in IRAK-4. Y105 appears to be unique for IRAK-M (Additional file 1: Figure S6D), and may be involved in the specific functionality of IRAK-M. Our protein docking simulations did not predict an interaction point of Y105 of IRAK-M with the death domains of IRAK-2 or IRAK-4, we speculate that potentially Y105 is involved in interaction of IRAK-M with other mediators such as MEKK3 or IRAK-1.

In conclusion, the present study presents a major improvement of our knowledge of the structure function relationships of the DD of IRAK-M. We have provided detailed information on the function of individual regions and amino acid residues in the IRAK-M DD and present an theoretical structural model that may integrate the currently known structure-function data of IRAK-M. Our findings may guide and support the targeting of the DD of human IRAK-M in efforts to generate treatment strategies to boost the host response in immunocompromised patients.

## Methods

### Homology modeling and interactive surface prediction

#### Template selection and alignment

The primary sequence of human IRAK-M consists of 596 amino acids. In order to determine the domain boundaries of the DD and in order to include the complete domain region, we applied multiple sequence analysis of IRAK-M from different species. The death domain could be modeled from C5 to G119 and potential templates for the IRAK-M DD were selected through PSI-BLAST of the protein data bank (PDB) [30]. PSI-BLAST (PSSM: 0.005) resulted in 12 available coordinate files, with sequence identities between 24% and 30% with the IRAK-M death domain sequence. Based on sequence alignment score and structure quality of potential template structures (resolution and R-factor) a final template was selected. We selected 2A9I (DD of mouse IRAK-4), [21] as a template, based on its sequence identity (28.7%). Moreover, 2A9I is a high resolution structure of 1.7 Å which was optimized by the PDB_REDO server with a structural quality of 0.619. The alignment between template and the IRAK-M sequence was performed by ICM-Pro (MolSoft) [31] with default scoring parameters and refined based on secondary structure prediction, amino acid features and the 3D structure of the template (Figure 1A and Additional file 2: Table S2).

#### Backbone generation and loop modeling

For the initial construction of the models we employed the ICM Pro molecular modeling package Molsoft as well as LOOPY [32]. ICM-Pro was used to generate the backbone coordinates for the models, from the refined sequence alignment between template and query structure, except in several variable (loop) regions, for which we used LOOPY. If the template and target had identical residues also the side chain coordinates could be included. Side chains were minimized by steepest descent and simulated annealing minimization with a fixed backbone conformation and next optimized without any restraint using the ICM Pro package.

#### MD simulation and quality check

The optimized model structure was further refined using a short MD simulation in explicit water (density 0.997, pH 7.0) for 500 picoseconds, employing the Yasara-Whatif twin package. The YAMBER3 force field was used, periodic boundaries and long range Coulomb interactions were included with a cutoff of 7.86Å. Every 25 ps, a simulation snapshot was saved, and in total, 20 snapshots were produced. Every snapshot and template were submitted for online structure quality check at http://nihserver.mbi.ucla.edu/SAVES_3/, using PROCHECK, WHATIF, VERIFY-3D, ERRAT and PROVE. The snapshot with the best total energy was selected as the starting point for a 100 ns MD simulation, with the AMBER03 force field periodic boundaries and long range Coulomb interactions were included with a cutoff of 7.86 Å in explicit water (Density 0.997, pH 7.0). The resulting energies, RMSD, residues' flexibility, hydrogen bonds and Ramachandran plots from the MD simulation were calculated.

Evaluation of the structural quality (Additional file 2: Table S3) of the final death domain model indicated that 92.1% of the residues were located in most favored zones and the remaining 7.9% were present in allowed regions as analyzed by inspection of a Ramachandran plot. Conformation Z-scores of both the model and the template were low, though the model’s side chain planarity and inter atomic distances were good. The structure’s non-bonding interactions were qualified as good in both the model and the template. The residue environment in the model is not better than that in template, but both of them are qualified as reasonable. A 100 ns MD simulation was performed to analyze the structural stability, amino acid fluctuation, and potential energy changes of the IRAK-M death domain (Additional file 1: Figure S1). During the MD simulation, the total energy (combined by bond, angle, planarity, coulomb, VdW) was stable while the conformation of the model changed during the simulation until 10 ns and then stabilized. The fluctuation of each residue during the MD simulation was calculated, and we observed that the loop between helix3 and helix4 was flexible. The number of hydrogen bonds, known to be important for protein stability and function [33], was generally consistent in the model during the simulation. A107 and N104 form hydrogen bonds with each other and thus connect helix 6 with the C-terminal loop.

#### Prediction of protein-protein contacts

A consensus approach was used to predict protein-protein interactions for the model structure generated by application of several structural bioinformatics methods. We employed: Optimal Docking Area (ODA) [34], Cons-PPISP [35] and PPI-Pred [36]. For each of these methods, a prediction score was obtained for all residues in the death domain of IRAK-M and a consensus was generated from the different methods applied, taking into account the accessibility of the residue. Several important residues for the interaction between the IRAK-4 DD and the DD of MyD88 have been predicted on basis of an earlier published model of the death domain: Q29, E92, F93, F94 at the surface of helix 2 and helix 5 [37]. Furthermore, residues which potentially interact with IRAK-1 in the N-terminus and the end of the helix 4: R12, C13, E69, D73, T76 were also predicted using PPI-Pred. T66 in the death domain of IRAK-1 is critical for interaction with signaling molecules reported by Neumann and coworkers [38]. In our model of the IRAK-M DD, the following residues in the homologous positions were found: C35, R47, E59, D63, T69, Q78, D85, R96, R97 respectively. Taking into account their flexibility and accessibility (Figure 1B), the residues, Q78, F18, D19, P21, P22, A23, W74, L20, R70, S75, R97, Y105, A77, L101, in order of likelihood, were predicted as potential sites of interaction with IRAK-M ligands.

Combined with residue spatial fluctuations as determined from MD simulation, hydrogen bond network analysis and homologue analysis, we proposed the mutagenesis of selected residues which we hypothesized to be involved in protein-protein interaction (Figure 1C).

### Mutagenesis and expression of human IRAK-M mutants

Human IRAK-M and IRAK-1 mammalian expression vectors (pUNO) containing a blasticidin resistance element were obtained from Invivogen (Toulouse, France). IRAK-M mutants were generated by site-directed mutagenesis with the QuickChange kit (Stratagene, La Jolla, CA) as recommended by the manufacturer with primers containing the desired mutations. Creation of an IRAK-M mutant which lacks the entire death domain was accomplished by introduction of a second SacII site (aaacta -> ccgcgg) in the ORF at codon position 103–105. SacII restriction of this mutated plasmid releases a fragment by cleavage at the introduced site and at the endogenous SacII site in codon 7–9 of the ORF. Ligation of the plasmid generates an ORF that encodes for amino acid 1–9 connected to 105–596 without introduction of additional amino acids. Constructs were subjected to DNA sequencing to confirm the mutations and check of the appropriate CDS. 293T cells, maintained in DMEM containing 10% FCS, were transfected using Lipofectamine 2000 (Invitrogen) as recommended by the manufacturer with the plasmids for protein expression and induction of NF-κB activation and IL-8 release essentially as described [1]. Lentiviral expression was obtained by introduction of WT IRAK-M in the pHEF vector and virus production in 293T cells as described [39,40]. The different IRAK-M Death Domain mutations were introduced in the constructed lentiviral human IRAK-M/IRES-eGFP expression system by restriction digestion of the pUNO-IRAK-M mutants with AgeI and BstB1 to obtain the mutated region which was ligated in the pHEF-IRAK-M vector which was digested with Xma1 (compatible overhang with Age1) and BstB1 to open up and remove the WT sequence. Non-replicating lentivirus production for transduction and expression of these mutants in cells was performed according to standard procedures [39]. Cell lines were transduced with the generated lentiviral constructs by standard procedures [40] with addition of polybrene. After lentiviral transduction with only eGFP as control, WT IRAK-M or the mutants, the populations of cells expressing the transgene were selected by cell sorting using the GFP signal as described [41].

The monocyte cell line THP-1 and transduced cultures were maintained in RPMI with 10% FCS and pen/strep. The bronchial type lung epithelial cell line H292, which is devoid of IRAK-M transcripts, was maintained as well as the transduced cultures in IMDM with 10% FCS and pen/strep.

### Western blotting

Westernblotting for IRAK-M was performed with 1 μg/ml monospecific rabbit anti-human IRAK-M antibodies directed against the C-terminal (Cell Signaling) or polyclonal mouse anti-full length human IRAK-M (Abnova) essentially as described [20]. B-actin antibodies were from Cell Signaling.

### NF-κB activation

Activation of NF-κB was determined 24 hours after transfection by cotransfection of a Firefly Luciferase NF-κB driven reporter construct and a Renilla Luciferase CMV driven construct on cell samples lysed with lysis buffer supplied with the DualGlo kit (Promega) used to determine the Firefly and Renilla luciferase activity in the same sample as recommended by the manufacturer.

### IL-8 expression

For IL-8 expression by transfected 293T cells, the cells were washed 24 hours after transfection and fresh culture medium was placed on the cells and supernatant was harvested 24 hours later and stored at −20°C for ELISA. Transduced H292 cells were plated in 96-wells cell culture plates (50.000 cells/well) and grown to confluency in 3 days and the medium was refreshed 24 hours before stimulation. Immediately before stimulation cells were washed again with medium again and cells were stimulated with 2 ng/ml IL-1β (Miltenyi Biotec) or 25 ng/ml Flagellin (Invivogen) for 6 hours and supernatant was collected and stored at −20°C for ELISA. IL-8 was determined using the duoset capture and biotinylated detecting antibody as recommended by the manufacturer (Invitrogen).

### TNF and IL-6

The monocyte cell line THP-1 and transduced cultures were maintained in RPMI with 10% FCS and pen/strep and matured to adherent macrophages and stimulated as described under conditions that will induce LPS tolerance after a previous exposure to LPS [42]. Supernatant of matured THP-1 cells stimulated with 1 ng/ml LPS (ultrapure, Invivogen) or 500 ng/ml Pam3CSK4 (Invivogen) for 6 hour was collected and stored at −20°C for determination of TNF and IL-6 by CBA (BD Biosciences) as described [43].

### IL-8, A20, SHIP and SOCS-3 mRNA expression

IL-8 and negative regulators of inflammation were determined at the mRNA level by RT-qPCR as described [20].

### The formation of IRAK-M-DD tetramer and the preparation of IRAK-4-DD tetramer, IRAK-2-DD tetramer and the complex of IRAK-2/IRAK-4/MyD88

Four IRAK-M-DD modules were superimposed onto either IRAK-2 (3D packing quality: −1.231) or IRAK-4 (3D packing quality: −1.241) DD tetramers respectively in the crystallographic model (3MOP, [3] by utilizing ICM-Pro Molsoft. The coordinates of four superimposed IRAK-M-DD were merged into one coordinate file of an IRAK-M-DD tetramer. The two obtained IRAK-M-DD tetramers were highly similar (Additional file 1: Figure S3A) with an all atom RMSD value of 1.05 Å. With the slightly higher 3D packing quality we used the IRAK-2-DD derived tetramer for further protein-protein docking studies. We first analyzed the atomic interactions (contact distance <=4 Å) between the individual IRAK-M monomers in the IRAK-M-DD tetramer (Additional file 1: Figure S3B). Next, IRAK-4 tetramer, IRAK-2 tetramers and IRAK-2/IRAK-4/MyD88 complex were retrieved from the crystallographic model 3MOP, with all hydrogen atoms added according to the protonation state at pH=7, which means that all acidic residues (Asp and Glu) are deprotonated and all basic residues (Lys and Arg) are protonated.

### Protein-protein docking

Hex protein docking [44] was applied to dock the IRAK-M-DD tetramer to IRAK-4-DD tetramer, IRAK-2-DD tetramer and IRAK-2/IRAK-4/MyD88 complex respectively. We analyzed 100 docking events for IRAK-4-DD tetramer binding to IRAK-M-DD and found that 63% of the docked poses were to the W74 exposing side of the IRAK-M-DD tetramer and 37% to the R97 exposing side of the IRAK-4-DD tetramer. The IRAK-M/IRAK4-DD complex was then docked with another IRAK-M-DD tetramer and the docked results showed that the sandwich IRAK-M/IRAK-4/IRAK-M complex was formed, where the W74 exposing site of IRAK-M tetramer docked with the bottom surface (IRAK-2 tetramer binding surface in 3MOP) of IRAK-4 tetramer and the R97 exposing site of IRAK-M tetramer docked with the top surface (MyD88 binding surface in 3MOP) of IRAK-4 tetramer.

Docking of the IRAK-M-DD tetramer to the IRAK-2-DD tetramer indicated that in a vast majority (83%) this would lead to a W74 exposing side (IRAK-M-DD tetramer) interaction and only in 17% in an interaction with the R97 exposing side. The IRAK-M-DD tetramer was docked to the bottom surface of the IRAK-2-DD tetramer from the IRAK-2/IRAK-4/MyD88 complex (Additional file 1: Figure S4F). The atomic interactions (contact distance <=4 Å) between IRAK-M-DD tetramer and the 3MOP structure were analyzed (Additional file 1: Figure S4E).

### Statistical analyses

Differences between WT and mutant protein were calculated by Mann–Whitney U test. Values are expressed as mean ± SEM. A p<0.05 was considered to represent a statistical significant difference.

## Additional files

Additional file 1: Figure S1. Analysis of 100 ns molecular dynamics (MD) simulation of hIRAK-M-DD model. **Figure S2.** Western blots on IRAK signaling proteins on lysates of 293T cells transfected with WT IRAK-M and mutants. No major differences were observed. **Figure S3.** **(A)** Superposition of the IRAK-M-DD tetramers modelled on the structure of the IRAK2-DD tetramer in 3MOP (gray) and IRAK-4-DD tetramer in 3MOP (Red). The 3D structure of those tetramers were highly similar and the all atom backbone root mean standard deviation (RMSD) between them was 1.05 Å. **(B)** IRAK-M-DD interaction points predicted to be important for homomeric tetramer formation based on the crystallographic model of human MyD88/IRAK-4/IRAK-2 complex (3MOP). The binding sites of one IRAK-M momomer (purple) to two other IRAK-M monomers (yellow and grey) in the IRAK-M tetramer. **Figure S4.** Stable expression of R70Q and D19N-A23S mutants in H292 cells as evaluated at the mRNA level by RT-qPCR. **Figure S5.** Effect of over expression of the different IRAK-M death domain mutants in H292 cells on the expression of other negative regulators. **Figure S6.** Comparison of the hIRAK-M Death Domain interactive surface with IRAK-2 and IRAK-4 DD’s. Additional file 2: Table S1. Predicted effect of IRAK-M death domain mutations on structural stability. **Table S2.** The structural quality of the template (2A9I from PDB_REDO *Joosten RP, Acta Cryst. 2012*) used to build human IRAK_M model. **Table S3.** Quality check of IRAK-M-DD model and the template by programs PROCHECK, WHATIF, VERIFY-3D, ERRAT and PROVE.
