# Supplementary material for: The structure function of the death domain of human IRAK-M
Source: Cell Commun Signal. 2014 Dec 7;12:77. doi: 10.1186/s12964-014-0077-3 (PMC4273448; doi:10.1186/s12964-014-0077-3)
Supplement: Additional file 1: Figure S1. — Analysis of 100 ns molecular dynamics (MD) simulation of hIRAK-M-DD model. Figure S2. Western blots on IRAK signaling proteins on lysates of 293T cells transfected with WT IRAK-M and mutants. No major differences were observed. Figure S3. (A) Superposition of the IRAK-M-DD tetramers modelled on the structure of the IRAK2-DD tetramer in 3MOP (gray) and IRAK-4-DD tetramer in 3MOP (Red). The 3D structure of those tetramers were highly similar and the all atom backbone root mean standard deviation (RMSD) between them was 1.05 Å. (B) IRAK-M-DD interaction points predicted to be important for homomeric tetramer formation based on the crystallographic model of human MyD88/IRAK-4/IRAK-2 complex (3MOP). The binding sites of one IRAK-M momomer (purple) to two other IRAK-M monomers (yellow and grey) in the IRAK-M tetramer. Figure S4. Stable expression of R70Q and D19N-A23S mutants in H292 cells as evaluated at the mRNA level by RT-qPCR. Figure S5. Effect of over expression of the different IRAK-M death domain mutants in H292 cells on the expression of other negative regulators. Figure S6. Comparison of the hIRAK-M Death Domain interactive surface with IRAK-2 and IRAK-4 DD’s. [file 12964_2014_77_MOESM1_ESM.pdf]

Additional file 1

Supplemental Figures

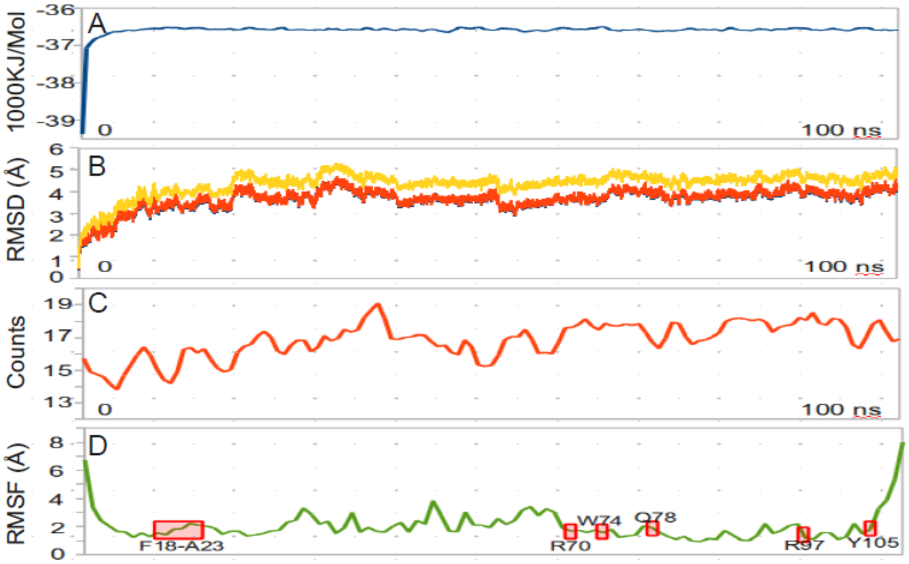

Figure S1. Analysis of 100 ns molecular dynamics (MD) simulation of hIRAK-M-DD model. **(A)** The total energy of DD during MD simulation. **(B)** RMS deviations of Ca (blue), backbone (red) and heavy chain (yellow). **(C)** The number of hydrogen bonds in DD during the simulation. **(D)** The backbone RMS fluctuation of each residue of DD was calculated, on the X-axis the residue number from R8 to P111. The predicted residues contributing to protein interaction were less flexible (RMSF ~ 2 Å) and are shown in the red boxes.

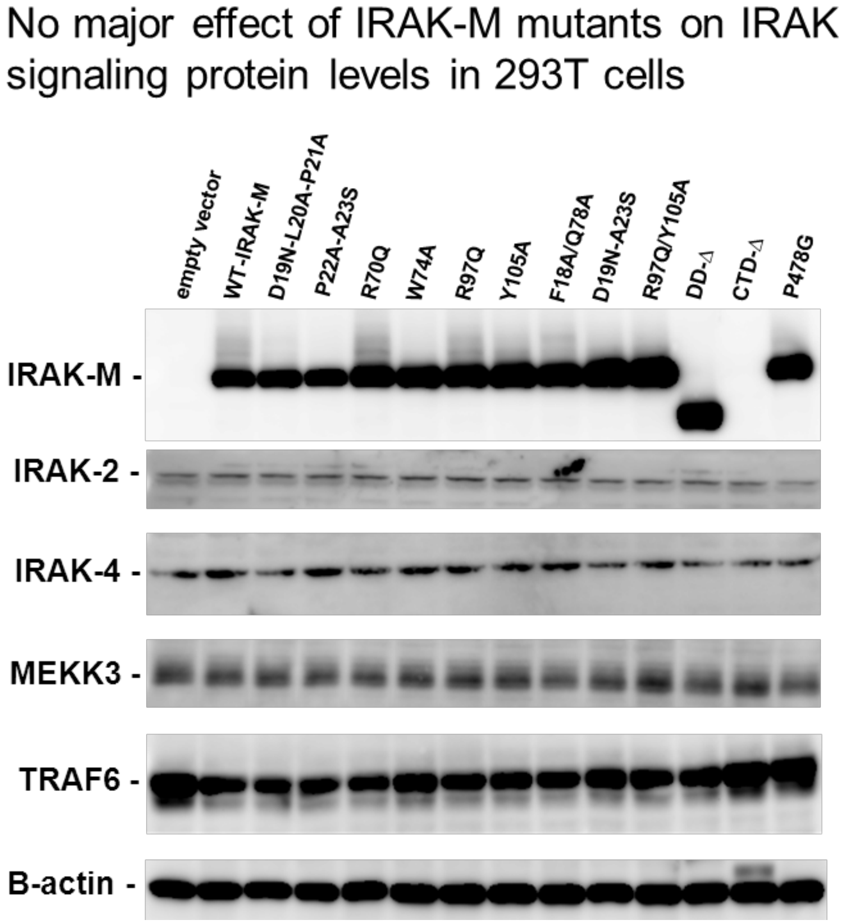

Figure S2. Western blots on IRAK signaling proteins on lysates of 293T cells transfected with WT IRAK-M and mutants. No major differences were observed

A B

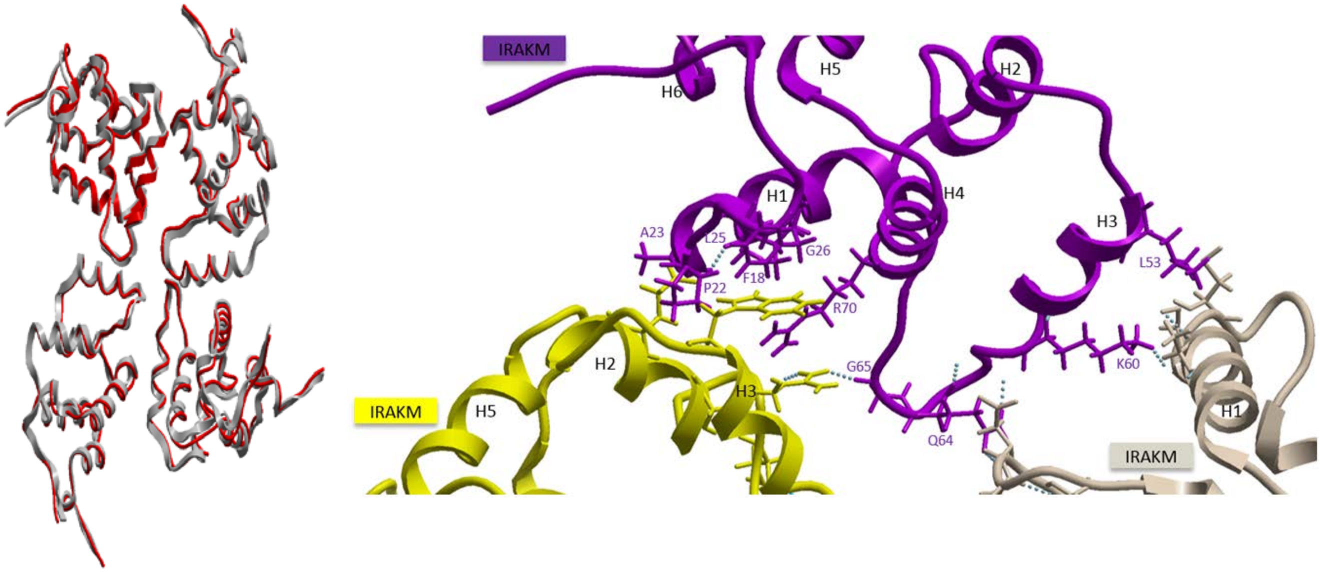

Figure S3. (A) Superposition of the IRAK-M-DD tetramers based on IRAK2-DD tetramer in 3MOP (gray) and IRAK-4-DD tetramer in 3MOP (Red). The 3D structure of those tetramers were highly similar and the all atom backbone root mean standard deviation (RMSD) between them was 1.05 Å. **(B)** IRAK-M-DD interaction points predicted to be important for homomeric tetramer formation based on the crystallographic model of human MyD88/IRAK-4/IRAK-2 complex (3MOP). The binding sites of one IRAK-M momomer (purple) to two other IRAK-M monomers (yellow and grey) in the IRAK-M tetramer.

A

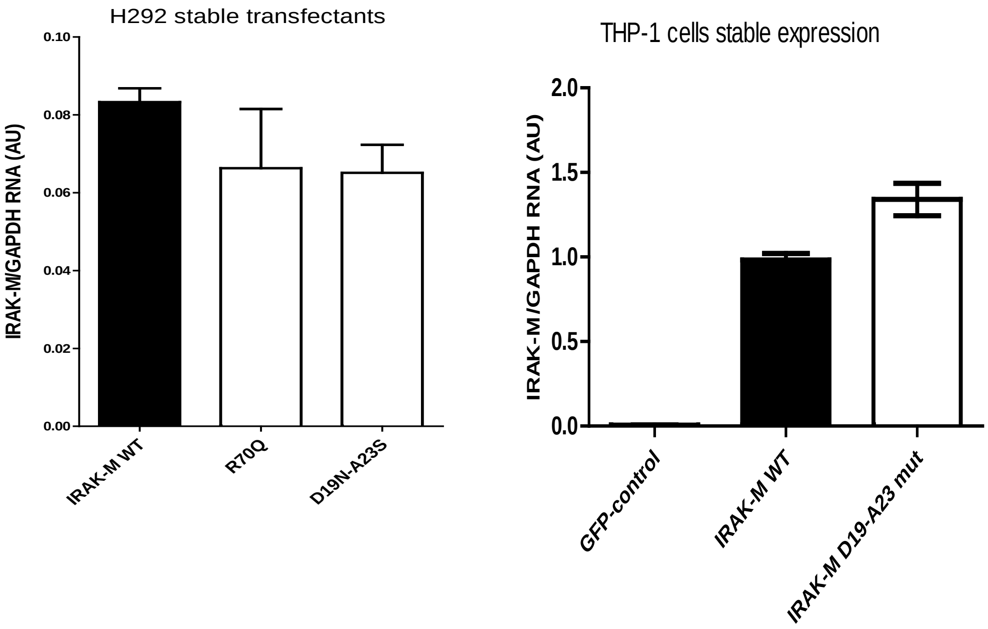

B

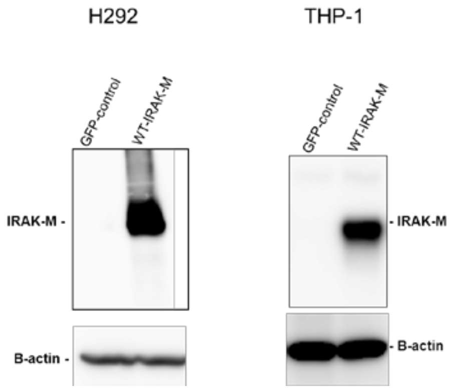

Figure S4. A) Stable expression of R70Q and D19N-A23S mutants in H292 cells and THP-1 cells as evaluated at the mRNA level by RT-qPCR. Mutants are transcribed equal to WT IRAK-M upon stable overexpression. The low observed protein levels upon stable expression in proficient cells is not due to lower transcription. B) Westernblot of WT IRAK-M stable expression in H292 and THP1 cells compared to GFP-control.

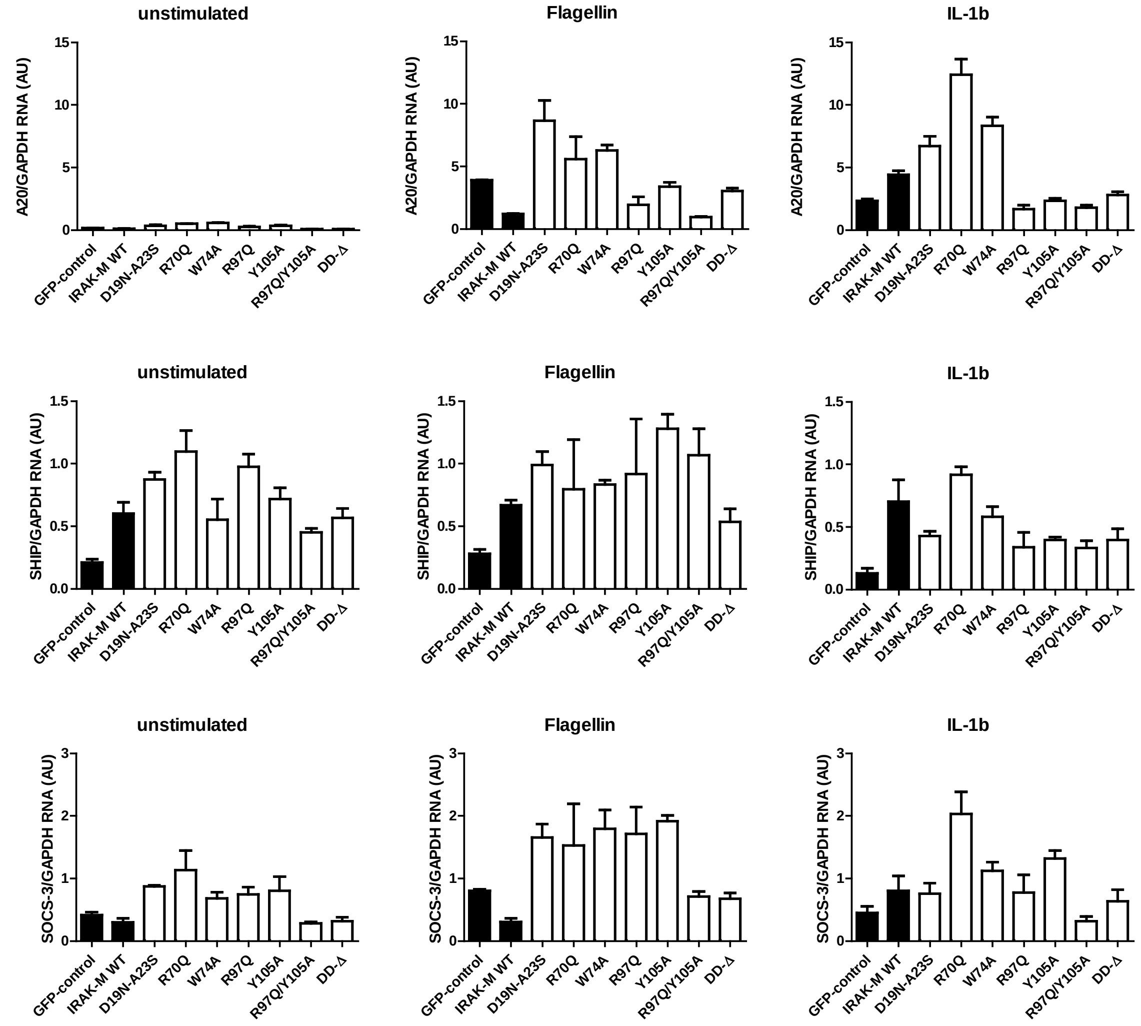

Figure S5. Effect of over expression of the different IRAK-M death domain mutants in H292 cells on the expression of other negative regulators. H292 cells were stimulated for 3 hours with Flagellin (25 ng/ml) or IL-1b (2 ng/ml) for 3 hours and mRNA expression was evaluated by RT-qPCR as described under materials and methods. N=3, mean±SEM.

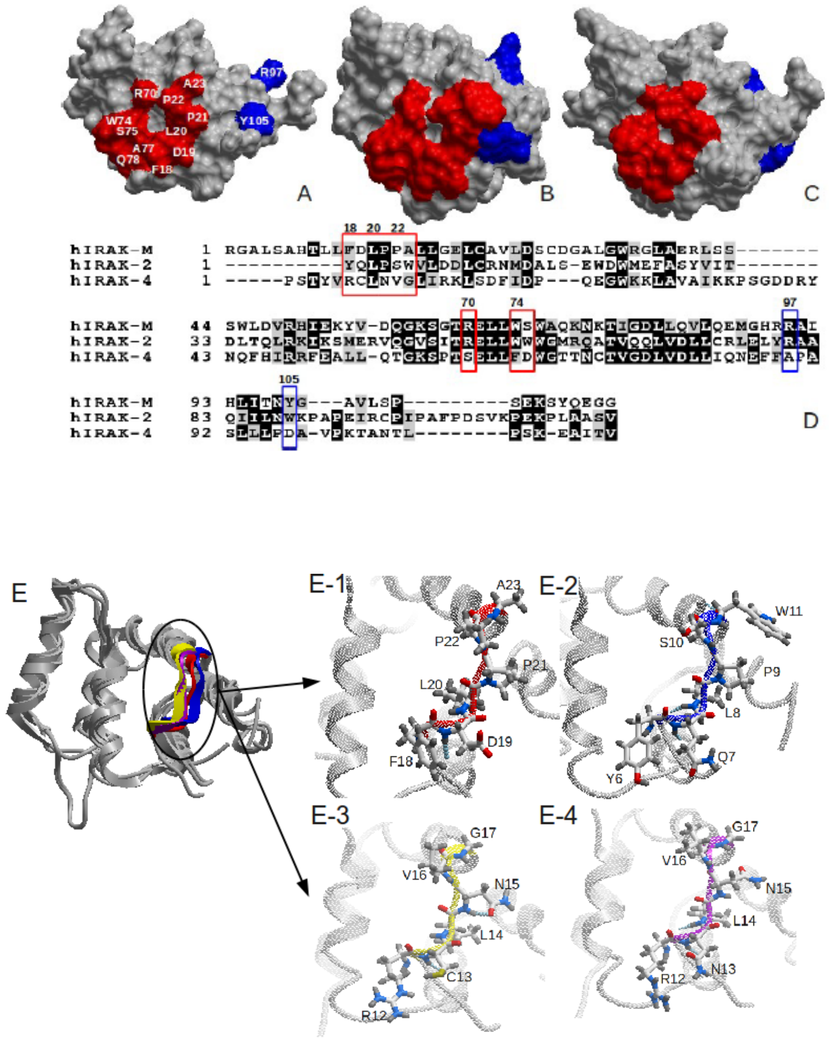

Figure S6. Comparison of the hIRAK-M Death Domain interactive surface with IRAK-2 and IRAK-4 DD's. Upper panel: Comparison of the defined interactive area's (red and blue) of the Death Domain of IRAK-M **(A)** to their homologous residues on human IRAK-2 **(B)** and human IRAK-4 **(C)**. Death Domains that were placed in a similar orientation by 3D structural overlay. **(D)** Multipe sequence alignments of the DD's shown in A-C. The residues involved in patch 1 and patch 2 are shown in red and blue boxes, respectively. **(E)** Superposition of IRAK members with the colored F19-A23 stretch. F18-A23 stretch and homologous residues shown in detail (E1-4). E-1:hIRAK-M, E-2:hIRAK-2, E-3:hIRAK-4, E-4:mIRAK-4.
