# Supplementary material for: The structure function of the death domain of human IRAK-M
Source: Cell Commun Signal. 2014 Dec 7;12:77. doi: 10.1186/s12964-014-0077-3 (PMC4273448; doi:10.1186/s12964-014-0077-3)
Supplement: Additional file 2: Table S1. — Predicted effect of IRAK-M death domain mutations on structural stability. Table S2. The structural quality of the template (2A9I from PDB_REDO Joosten RP, Acta Cryst. 2012) used to build human IRAK_M model. Table S3. Quality check of IRAK-M-DD model and the template by programs PROCHECK, WHATIF, VERIFY-3D, ERRAT and PROVE. [file 12964_2014_77_MOESM2_ESM.pdf]

Additional file 2, Supplemental Tables

Table S1. Predicted effect of the mutations in IRAK-M death domain

Residue substitution	Change in solvent accessibility	Pseudo ΔΔ G (kcal mol-1)	Predicted effect on protein stability
F18A	-12.10%	2.36	No effect
D19N-L20A-P21A	38.7%	-0.5	No effect
P22A-A23S	14.6%	2.16	No effect
R70Q	-2.4%	-0.26	No effect
W74A	-12.1%	2.36	No effect
Q78A	4.3%	0.99	No effect
F18A-P21A	26.6%	1.86	No effect
D19N-A23S	53.3%	1.66	No effect
F18A/Q78A	-8.1%	3.35	No effect
R97Q	-8.3%	-0.07	No effect
Y105A	-1.6%	2.22	No effect
R97Q/Y105A	-9.9%	2.15	No effect

Predicted effect of IRAK-M death domain mutations on structural stability.
The mutations studied in the present include 11 individual residues: F18, D19, L20, P21, P22, A23, R70, W74, Q78, R97, and Y105. The 3D model of the wild type IRAK-M death domain was *in silico* mutated at these 11 residues by the YASARA/WhatIf twinpackage, followed by a 3 nanoseconds molecular dynamic simulation with the yasara2 force field in water to optimize the structure. The mutated model structures were next evaluated by the online server SDM (http://www-cryst.bioc.cam.ac.uk/~sdm/sdm.php) to predict the structural stability by the mutation [6]. This method applies a statistical potential energy function to calculate the pseudo ΔΔ free energy by using properties such as environment-specific amino acid substitution frequencies from the targeted protein homologous families. This value is comparable to the free energy difference between wild type and the mutant. From the server, five parameters: secondary structure of the mutated residue, solvent accessibility changes, hydrogen bond changes, pseudo ΔΔG and predicted effect on protein stability are generated for each single amino acid mutation. The solvent accessibility changes were the difference of the solvent accessibility (%) of the mutated residue and the wild type residue. F18A and W74A lost around 12% of the solvent accessible area and highly stabilized the structures while D19N and P21A gained 17.7% and 21.2% of the solvent accessible area.

The predicted effect of the multiple mutations on structural stability and the change in solvent accessibility of multiple mutations were calculated by the sum of the values from corresponding individual mutation values. The mutants with which experiments were performed in our work are shown in table S1. These mutants were predicted to stabilize or did not affect the death domain structure. Meanwhile, the multiple mutants D19N-L20A-P21A, P22A-A23S, F18A-P21A, D19N-A23S increased the solvent accessible area.

Table S2. The structural quality of the template (2A9I from PDB_REDO *Joosten RP, Acta Cryst. 2012*) used to build human IRAK_M model.

R Value	0.17	Backbone conformation[1]	-0.82
1st packing[1]	-0.81	Bond length RMS Z-score[2]	0.48
2nd packing[1]	-1.02	Bond angle RMS Z-score[2]	0.70
Ramachandran	0.82	Total number of bumps[3]	8
Chi-1/Chi-2[1]	-0.18	Unsatisfied H-bond donors/acceptors[3]	3

[1] Higher is better; [2] Should be lower than 1; [3] Fewer is better; Full WHAT_CHECK results of the template can be viewed in http://www.cmbi.ru.nl/pdb_redo/a9/2a9i/wf/index.html.

Table S3. Quality check of IRAK-M-DD model and the template by programs PROCHECK, WHATIF, VERIFY-3D, ERRAT and PROVE .

	IRAK-M-DD Model	Template (2a9i)
RMSD (Å)[1]	1.88	
3D Packing Quality[2]	-0.38	-0.07
Surface Area ($Å^2$)	7037.9	6987
Ramachandran abnormal	L39	-
Chi1-Chi2 abnormal	Y61 W76	L60
Planarity abnormal	W41	D73
Bump	G65-K66	-
1D-3D consensus[2]	87.38%	96.19%
Errat[2]	99.00%	100.00%
PROVE z-score[2]	0.4 (-5, 5)	0.153 (-5, 5)

[1] The backbone root mean square distance between IRAK-M-DD and the template. [2] The higher the better. Errat is to analyze the non-bonded interactions in protein 3D structures and generate a confidence limits (0-1) to judge reliability of a protein's 3D structure [4]. PROVE is to check the atomic volume [5] and calculate the RMSD z-score between a given protein structure and the PDB dataset. The ideal PROVE z-score is expected as 0. A negative z-score means the atom volume smaller than average while a positive z-score means the atom volume greater than average.

1. Hooft, R.W., et al., *Errors in protein structures.* Nature, 1996. **381**(6580): p. 272.
2. Lin, S.C., Y.C. Lo, and H. Wu, *Helical assembly in the MyD88-IRAK4-IRAK2 complex in TLR/IL-1R signalling.* Nature, 2010. **465**(7300): p. 885-90.
3. Ghoorah, A.W., et al., *Protein docking using case-based reasoning.* Proteins, 2013.
4. Colovos, C. and T.O. Yeates, *Verification of protein structures: patterns of nonbonded atomic interactions.* Protein science : a publication of the Protein Society, 1993. **2**(9): p. 1511-9.
5. Pontius, J., J. Richelle, and S.J. Wodak, *Deviations from standard atomic volumes as a quality measure for protein crystal structures.* Journal of molecular biology, 1996. **264**(1): p. 121-36.
6. Worth, C.L., R. Preissner, and T.L. Blundell, *SDM--a server for predicting effects of mutations on protein stability and malfunction.* Nucleic acids research, 2011. **39**(Web Server issue): p. W215-22.
